# Comparison of nasal and oral linear endobronchial ultrasound: a retrospective single-center study

**DOI:** 10.1186/s12890-026-04389-7

**Published:** 2026-06-17

**Authors:** Aydın Balcı, Suphi Aydın, Yaşar İnkaya, Yunus Ardıç, Şule Çilekar, İbrahim Güven Çoşğun, Ersin Günay

**Affiliations:** 1 Department of Pulmonology, Afyonkarahisar Health Sciences University, Afyonkarahisar, Turkey; 2 Department of Thoracic Surgery, Health Sciences University, Ankara, Turkey; 3 Department of Pulmonology, Ankara City Hospital, Ankara, Turkey

**Keywords:** Endobronchial ultrasound, EBUS-TBNA, Lung cancer, Mediastinal lymphadenopathy, Nasal bronchoscopy, Oral bronchoscopy

## Abstract

**Background:**

Endobronchial ultrasound-guided transbronchial needle aspiration (EBUS-TBNA) is a safe and effective technique for lung cancer staging and evaluation of mediastinal and hilar lesions. Although oral insertion is widely used, the nasal route may improve scope stability and patient tolerance, but comparative data remain limited.

**Methods:**

This retrospective single-center study included adults who underwent linear EBUS-TBNA for mediastinal or hilar lymphadenopathy between January 2019 and December 2023. Patients were grouped according to insertion route. Standardized topical anesthesia, conscious sedation, procedural monitoring, and post-procedural recovery criteria were used. Hemodynamic parameters, oxygen saturation, sedative and analgesic requirements, lymph node sampling, diagnostic results, complications, and post-procedural complaints were compared between the nasal and oral groups.

**Results:**

Nasal EBUS was performed in 158 patients (39.1%) and oral EBUS in 246 patients (60.9%). Baseline and intraprocedural oxygen saturation were similar between groups, whereas post-procedure oxygen saturation was higher in the nasal group. Oral EBUS was associated with a greater intraprocedural heart-rate increase, higher midazolam and propofol requirements, more frequent hypoxemia-related procedure interruption, and a higher re-EBUS rate. The nasal route was associated with modestly shorter procedures and slightly higher numbers of sampled lymph node stations and punctures per examination. Diagnostic categories and overall complication rates were comparable between groups.

**Conclusion:**

In this large single-center retrospective cohort, nasal linear EBUS-TBNA was a feasible and safe alternative to oral EBUS-TBNA. The nasal route provided comparable diagnostic performance and complication rates while reducing sedative requirements, hypoxemia-related interruptions, and procedure time. These findings support the nasal route as a practical option for interventional pulmonology units experienced with linear EBUS.

## Background

Endobronchial ultrasound (EBUS) is a safe and effective technique for lung cancer staging and evaluation of mediastinal and hilar lesions. EBUS-guided transbronchial needle aspiration (EBUS-TBNA) enables real-time sampling of mediastinal and hilar lymph nodes with high diagnostic yield and a low complication rate [1–3].

Oral insertion is the most commonly preferred route for linear EBUS because the distal end of the EBUS bronchoscope is relatively large and firm. The oral route usually requires a mouthpiece and continuous manual stabilization of the bronchoscope. In selected centers, the nasal route is used to increase bronchoscope stability, reduce oral secretion and coughing, and improve procedural tolerance [4–7]. However, comparative evidence regarding the safety, feasibility, diagnostic performance, and procedure-related outcomes of nasal versus oral linear EBUS remains limited.

During the COVID-19 pandemic, our unit began to use nasal EBUS more frequently to reduce oral manipulation, secretion burden, and suctioning during procedures. In our experience, the nasal route provided stable positioning of the bronchoscope without damage to the EBUS device. Rigid bronchoscopy was not used in either the nasal or oral EBUS procedures included in the present cohort; all procedures were performed with flexible linear EBUS bronchoscopes.

The present study aimed to compare nasal and oral linear EBUS-TBNA with respect to procedural safety, sedative requirements, hemodynamic and oxygenation parameters, diagnostic results, complications, and post-procedural complaints.

## Materials and methods

### Study design, setting, and data sources

This retrospective single-center study evaluated consecutive patients who underwent linear EBUS-TBNA for mediastinal or hilar lymphadenopathy at Afyonkarahisar Health Sciences University between January 2019 and December 2023. The study was conducted in accordance with the principles of the Declaration of Helsinki. Ethical approval was obtained from the Afyonkarahisar Health Sciences University Clinical Research Ethics Committee (approval no. 2023/573; 15 December 2023). Because of the retrospective design, the ethics committee waived the requirement for informed consent for data analysis. Written informed consent for the bronchoscopy procedure itself was obtained from all patients as part of routine clinical practice.

Data were extracted from the electronic medical records, bronchoscopy reports, anesthesia records, procedure monitoring forms, pathology reports, and follow-up notes. Recorded variables included age, sex, smoking status, comorbidities, procedure indication, insertion route, topical anesthesia and sedative/analgesic drugs, hemodynamic parameters, oxygen saturation, sampled lymph node stations, number of punctures, procedure duration, pathology result, complications, procedure interruptions, repeat EBUS procedures, and post-procedural complaints documented within 24 h.

### Eligibility criteria and grouping

Patients aged 18 years or older who underwent linear EBUS-TBNA and had complete procedural and pathology records were eligible. Cases were excluded from the analysis if key procedural details were missing, if EBUS-TBNA was combined with another bronchoscopic intervention that could affect procedure duration or complications, or if the procedure was cancelled before lymph node sampling. Patients with standard institutional contraindications to EBUS-TBNA or sedation, including clinically significant bleeding diathesis, uncontrolled severe cardiopulmonary instability, severe active lower respiratory tract infection, pregnancy, severe neuropsychiatric conditions preventing safe sedation, allergy to planned anesthetic or analgesic drugs, or failure to discontinue antiplatelet or anticoagulant therapy when required, were not included. Because the study was retrospective, these criteria were applied during chart review and data extraction rather than by prospective patient selection.

Patients were classified according to the insertion route documented in the bronchoscopy report: nasal EBUS-TBNA and oral EBUS-TBNA. The number of patients in each group and baseline characteristics are presented in the Results section and Table 1.

**Table 1:** Comparative analysis of demographic characteristics, monitoring parameters, and sedation outcomes

Parameter	Group 1: nasal EBUS (*n* = 158)	Group 2: oral EBUS (*n* = 246)	*p*-value
Age, years (mean ± SD)	60.52 ± 12.08	60.36 ± 13.41	0.900
Female, n (%)	62 (39.2%)	88 (35.8%)	0.480
Male, n (%)	96 (60.8%)	158 (64.2%)	
Smoking status, n (%)			0.042
Smoker	61 (38.6%)	109 (44.3%)	
Non-smoker	57 (36.1%)	99 (40.2%)	
Former smoker	40 (25.3%)	38 (15.4%)	
HADS-A psychopathology	12 (7.6%)	13 (5.1%)	0.620
HADS-A borderline	29 (18.4%)	51 (20.7%)	0.570
HADS-A no psychopathology	117 (74.1%)	182 (74.0%)	0.708
Comorbidity, n (%)	84 (53.2%)	118 (48.0%)	0.309
Heart rate T1, beats/min	93.72 ± 14.51	93.20 ± 14.76	0.790
Heart rate T2, beats/min	103.77 ± 19.63	105.25 ± 21.48	0.023
Heart rate T3, beats/min	100.31 ± 15.29	104.30 ± 19.23	0.029
SpO2 T1, %	94.18 ± 7.07	94.36 ± 2.16	0.389
SpO2 T2, %	90.08 ± 8.94	89.37 ± 7.08	0.515
SpO2 T3, %	91.52 ± 4.86	89.95 ± 6.93	0.010
Ramsay score 4	104 (65.8%)	21 (8.5%)	0.010
Ramsay score 5	12 (7.6%)	17 (6.9%)	
Ramsay score 6	42 (26.6%)	208 (84.6%)	
Midazolam, mg	3.53 ± 0.94	4.09 ± 0.87	0.027
Propofol, mg	192.91 ± 66.65	251.34 ± 83.98	0.001
Alfentanil, mg	1.32 ± 0.71	1.44 ± 0.57	0.070

*EBUS* endobronchial ultrasound, *HADS-A* Hospital Anxiety and Depression Scale-Anxiety subscale, *SD* standard deviation, *SpO2* peripheral oxygen saturation. T1, pre-procedure baseline; T2, intraprocedural value; T3, post-procedure recovery value

### EBUS-TBNA procedure and equipment

All procedures were performed by an experienced pulmonologist with more than three years of EBUS experience and an assisting pulmonologist. An anesthesia technician and a bronchoscopy nurse were present throughout the procedure. Linear EBUS-TBNA was performed with a convex-probe ultrasound bronchoscope (BF-UC180F, Olympus, Tokyo, Japan). Ultrasound images were obtained using an EU-ME1 ultrasound processor (Olympus, Tokyo, Japan) or a Prosound Alpha10 ultrasound system (Aloka, Tokyo, Japan), according to device availability. Ultrasonographic evaluation was performed in B-mode and color Doppler mode before puncture to identify vascular structures and avoid vascular injury. A 22-gauge EBUS-TBNA needle (NA-201SX-4022, Olympus, Tokyo, Japan) was used for sampling. A balloon was not used routinely; it was applied only when acoustic contact was insufficient according to operator judgment.

Before nasal insertion, nasal patency was assessed clinically, and the more patent nostril was selected. The bronchoscope was lubricated and advanced gently along the nasal floor under direct visualization. When needed, the distal end was rotated approximately 90 degrees during passage through the nasal cavity and then returned to the standard orientation after entry into the nasopharynx. Forceful insertion was avoided, particularly in patients with narrow nasal passages or a history suggestive of recent epistaxis. If nasal insertion caused uncontrolled pain, clinically relevant bleeding, or inability to advance the bronchoscope safely, the route was converted to oral insertion with a mouthpiece.

For oral EBUS, the bronchoscope was introduced through a protective mouthpiece and stabilized manually by the bronchoscopist and assistant. For nasal EBUS, the nasal alae provided additional natural fixation of the scope, and the operator maintained the final position manually. After airway inspection and ultrasonographic localization, bilateral paratracheal, subcarinal, hilar, and interlobar lymph node stations were systematically evaluated when clinically indicated. The target lymph node was punctured under real-time ultrasound guidance. At least three needle passes were planned per lymph node station whenever feasible. Aspirated samples were placed in formaldehyde and sent for cytopathologic and/or histopathologic evaluation according to routine institutional practice.

### Topical anesthesia, sedation, and monitoring

All procedures were performed under conscious sedation with spontaneous breathing. For oral EBUS, topical anesthesia was applied to the oropharynx and larynx. For nasal EBUS, 2.5 mL of 2% viscous lidocaine was applied to each nostril, and 4% lidocaine solution or lidocaine aerosol spray was applied to the oropharynx.

Sedation was initiated and titrated according to institutional protocols and patient age, body weight, comorbidity profile, and procedural response. Midazolam was used at 0.03–0.05 mg/kg in patients younger than 60 years without major comorbidity and at approximately 0.015 mg/kg in older patients or those with comorbidities. Alfentanil was used as the opioid analgesic; fentanyl was not part of the routine protocol in this cohort. Propofol was administered as a small initial bolus, generally 0.5-1 mg/kg, followed by titrated boluses as needed to maintain adequate comfort and procedural conditions. The target depth of sedation was a Ramsay Sedation Scale score of at least 4 [8].

All patients received supplemental oxygen via a face mask during the procedure. Ambu-bag ventilation was used only as rescue support when clinically necessary, such as during clinically significant hypoxemia or inadequate spontaneous ventilation. Heart rate, blood pressure, and pulse oximetry were monitored continuously or at short intervals according to routine anesthesia practice.

### Outcome measures and definitions

The primary comparisons were procedural feasibility and safety of nasal versus oral EBUS-TBNA. Secondary comparisons included sedative and analgesic doses, oxygenation and heart-rate changes, procedure duration, lymph node stations sampled, punctures per examination, diagnostic categories, complications, and post-procedural complaints.

Oxygen saturation (SpO2) and heart rate were recorded at three predefined time points. T1 was the pre-procedure baseline value measured after monitoring and supplemental oxygen setup, before bronchoscope insertion. T2 was the intraprocedural value recorded during EBUS-TBNA; when multiple values were documented, the lowest SpO2 and the corresponding or clinically most relevant heart-rate value were used. T3 was the post-procedure recovery value documented after bronchoscope removal and clinical stabilization before discharge from the bronchoscopy unit or transfer back to the ward.

Hypoxemia was defined as SpO2 below 88% for longer than 30 s. Persistent hypoxemia was defined as hypoxemia that did not resolve promptly with simple maneuvers and required bronchoscope withdrawal, procedure interruption, assisted ventilation, or termination of the procedure. Hypotension was defined as systolic blood pressure below 90 mmHg, mean arterial pressure below 65 mmHg, or a decrease of more than 20% from baseline requiring clinical intervention. Laryngospasm and bronchospasm were diagnosed clinically by airway resistance, wheezing or stridor, auscultation, and response to bronchodilator or airway maneuvers. Re-EBUS was defined as the need to repeat EBUS-TBNA because the initial procedure could not be completed due to hypoxemia or procedure interruption.

### Post-procedural observation

After the procedure, patients were monitored in the recovery area until they met institutional discharge criteria. Recovery was assessed using the Aldrete recovery score, and patients were discharged from the bronchoscopy unit or transferred back to the ward when the score reached at least 9 and the responsible clinician considered the patient clinically stable [9]. Outpatients were observed for at least two hours. Inpatients returned to the relevant ward after recovery. Complications occurring within the first 24 h were recorded from bronchoscopy, ward, and emergency department notes.

### Statistical analysis

Statistical analyses were performed using SPSS for Windows, version 25.0 (IBM Corp., Armonk, NY, USA). Continuous variables were summarized as means and standard deviations. Categorical variables were summarized as counts and percentages. Normality was assessed using the Kolmogorov-Smirnov test. Between-group comparisons of normally distributed continuous variables were performed with Student’s t-test, and non-normally distributed continuous variables were compared using the Mann-Whitney U test. Categorical variables were compared using the chi-square test or Fisher’s exact test, as appropriate. A two-sided p-value below 0.05 was considered statistically significant. Because of the retrospective design and incomplete weight-adjusted dosing records for some patients, sedative doses were analyzed as total administered doses rather than dose per kilogram per minute.

## Results

A total of 404 patients were included. Nasal EBUS was performed in 158 patients (39.1%; group 1), and oral EBUS was performed in 246 patients (60.9%; group 2). Overall, 254 patients (62.9%) were male and 150 (37.1%) were female. There was no significant difference in sex distribution between the groups (*p* = 0.480). Mean age was similar in the nasal and oral groups (60.52 ± 12.08 years vs. 60.36 ± 13.41 years, *p* = 0.900). Smoking status differed between groups (*p* = 0.042), mainly because of a higher proportion of former smokers in the nasal group. HADS-A categories and comorbidity rates did not differ significantly between groups (Table 1).

SpO2 values at T1 and T2 were not significantly different between groups, whereas T3 SpO2 was significantly higher in the nasal group (91.52 ± 4.86% vs. 89.95 ± 6.93%, *p* = 0.010). Baseline heart rate was similar between groups. Heart rate was higher in the oral group during the procedure (T2, *p* = 0.023) and at recovery measurement (T3, *p* = 0.029). Ramsay Sedation Scale scores differed between groups, with deeper sedation levels more frequent in the oral group. The mean midazolam and propofol doses were significantly higher in the oral group, whereas alfentanil doses were similar between groups (Table 1).

Procedure indications were similarly distributed between groups. The nasal group had a modestly shorter mean procedure time (33.27 ± 11.12 min vs. 35.95 ± 11.16 min, *p* = 0.019), a slightly higher mean number of sampled lymph node stations (2.47 ± 1.26 vs. 2.20 ± 1.02, *p* = 0.017), and a slightly higher mean number of punctures per examination (4.44 ± 1.22 vs. 4.12 ± 0.95, *p* = 0.003). Hypoxemia was more common in the oral group (24.4% vs. 15.2%, *p* = 0.026), and re-EBUS due to hypoxemia-related interruption was also more common in the oral group (9.3% vs. 1.9%, *p* = 0.003). Post-procedure jaw discomfort or anterior toothache was reported predominantly in the oral group (25.2% vs. 0.6%, *p* < 0.001). Bronchial bleeding, nasal bleeding, and pneumothorax rates did not differ significantly between groups (Table 2).

**Table 2:** Comparative analysis of procedural outcomes and complications

Parameter	Total (*n* = 404)	Group 1: nasal EBUS (*n* = 158)	Group 2: oral EBUS (*n* = 246)	*p*-value
Indication: lung cancer staging, n (%)	74 (18.3%)	28 (17.7%)	46 (18.7%)	0.805
Indication: diagnosis, n (%)	330 (81.7%)	130 (82.3%)	200 (81.3%)	
Procedure time, min (mean ± SD)	-	33.27 ± 11.12	35.95 ± 11.16	0.019
Lymph node stations sampled (mean ± SD)	-	2.47 ± 1.26	2.20 ± 1.02	0.017
Punctures per examination (mean ± SD)	-	4.44 ± 1.22	4.12 ± 0.95	0.003
Hypoxemia, n (%)	84 (20.8%)	24 (15.2%)	60 (24.4%)	0.026
Re-EBUS due to hypoxemia, n (%)	26 (6.4%)	3 (1.9%)	23 (9.3%)	0.003
Post-procedure jaw discomfort/anterior toothache, n (%)	63 (15.6%)	1 (0.6%)	62 (25.2%)	< 0.001
Bronchial bleeding, n (%)	8 (1.9%)	2 (1.3%)	6 (2.4%)	0.107
Pneumothorax, n (%)	0	0	0	1.000
Nasal bleeding, n (%)	8 (1.9%)	2 (1.3%)	6 (2.4%)	0.107

*EBUS* endobronchial ultrasound, *SD* standard deviation

The distribution of final diagnostic categories did not differ significantly between groups (*p* = 0.104). Malignancy was identified in 41 patients (25.9%) in the nasal group and 71 patients (28.9%) in the oral group. Granulomatous disease, lymphoproliferative disease, reactive lymph nodes, and non-diagnostic samples were similarly distributed (Table 3). No procedure-related death or permanent organ dysfunction was recorded.

**Table 3:** Distribution of final diagnostic categories

Diagnosis	Total (*n* = 404)	Group 1: nasal EBUS (*n* = 158)	Group 2: oral EBUS (*n* = 246)	*p*-value
Malignancy, n (%)	112 (27.7%)	41 (25.9%)	71 (28.9%)	0.104
Granulomatous disease, n (%)	51 (12.6%)	18 (11.4%)	33 (13.4%)	
Lymphoproliferative disease, n (%)	7 (1.7%)	1 (0.6%)	6 (2.4%)	
Reactive lymph node / negative for malignancy, n (%)	232 (57.4%)	98 (62.0%)	134 (54.5%)	
Non-diagnostic / inadequate sample, n (%)	2 (0.5%)	0 (0%)	2 (0.8%)	

*EBUS* endobronchial ultrasound

## Discussion

In this retrospective single-center study of 404 patients undergoing linear EBUS-TBNA, the nasal route was feasible and safe and provided diagnostic results comparable to those of the oral route. The main clinically relevant findings were lower sedative requirements, less hypoxemia-related interruption, fewer re-EBUS procedures, and a modest reduction in procedure time in the nasal group. These findings suggest that route selection may influence the technical conduct of EBUS-TBNA even when diagnostic categories and major complication rates remain similar.

The feasibility of nasal linear EBUS has been reported previously, but available studies remain limited. Beaudoin et al. showed that nasal placement of a linear EBUS bronchoscope was possible in most patients and did not increase complications. A later randomized study found that the oral and nasal routes were both acceptable with respect to patient comfort. Piro et al. also reported comparable safety and diagnostic accuracy for the transnasal approach. Our results are generally consistent with these studies, but our cohort adds a relatively large single-center experience and highlights clinically practical outcomes such as hypoxemia-related interruption and re-EBUS frequency [5–7].

The lower rate of hypoxemia and hypoxemia-related re-EBUS in the nasal group may be explained by several mechanisms. First, nasal insertion leaves the oral cavity more available for oxygen delivery, airway maneuvers, and rescue ventilation. Second, the nasal alae can provide additional stabilization of the bronchoscope, reducing repeated repositioning. Third, oral procedures may require deeper sedation to tolerate the mouthpiece and oral manipulation. In the present study, the oral group received higher total midazolam and propofol doses and had more frequent deeper Ramsay scores, which may have contributed to respiratory compromise. Because drug dosing was evaluated as total dose rather than weight- and time-adjusted dose, this interpretation should be considered hypothesis-generating rather than definitive. Previous studies comparing anesthesia and sedation strategies for EBUS-TBNA also support the importance of balancing procedural comfort with avoidance of excessive respiratory depression [10, 11].

The differences in procedure time, number of sampled stations, and number of punctures were statistically significant but numerically modest. A mean difference of approximately 2.6 min may not be meaningful for an individual patient, but it can become relevant in high-volume bronchoscopy units, especially when combined with fewer procedure interruptions and fewer repeat procedures. Similarly, the higher number of sampled stations and punctures in the nasal group was small; therefore, the finding should not be interpreted as proof of superior diagnostic performance. Rather, it suggests that improved scope stability may facilitate completion of the planned sampling strategy in some cases.

Diagnostic category distribution was comparable between groups. This supports the main practical message of the study: nasal EBUS can be used without compromising diagnostic performance when performed by an experienced team. EBUS-TBNA is already established as a first-line method for mediastinal staging and diagnosis because of its high yield and favorable safety profile. Current technical recommendations emphasize systematic evaluation, real-time ultrasound guidance, Doppler assessment to avoid vascular puncture, and an adequate number of needle passes per target lymph node station [12–14]. Our procedural protocol was consistent with these principles.

Major complications were uncommon in both groups. Bleeding and nasal bleeding rates did not differ significantly, which is important because epistaxis is a common concern when considering the nasal route. Careful nostril selection, lubrication, gentle insertion along the nasal floor, avoidance of forceful passage, and conversion to the oral route when needed are likely important for minimizing nasal trauma. No pneumothorax was observed in either group. The low overall complication profile is consistent with large bronchoscopy and EBUS registry data [15, 16].

Post-procedure jaw discomfort and anterior toothache were more frequently documented after oral EBUS. We did not use neuromuscular blocking agents, and these complaints should not be interpreted as masseter muscle injury caused by succinylcholine or other paralytic drugs. The most plausible explanation is mechanical stress related to prolonged mouth opening, mouthpiece pressure, protective oral devices, and occasional jaw clenching during conscious sedation. Although most complaints were transient, the high frequency in the oral group indicates that post-procedural oral and temporomandibular discomfort should be actively recorded in future prospective studies using standardized patient-reported outcome measures. Patient comfort should be interpreted in the context of prior bronchoscopy studies showing that coughing and procedure-related discomfort can substantially influence the patient experience [17].

Our findings also have practical implications for bronchoscopy workflow. Oral EBUS frequently requires continuous suctioning and close manual stabilization of the bronchoscope. The nasal route may reduce oral secretion burden and make ventilation maneuvers easier during transient desaturation. However, these potential advantages should be balanced against patient anatomy, nasal patency, bleeding risk, operator experience, and device availability. This may also be relevant for device handling and repair costs, which have been highlighted as an important practical issue in EBUS services [18].

This study has several limitations. First, the retrospective design creates a risk of selection bias and residual confounding. Second, route allocation was not randomized and may have been influenced by operator preference, patient anatomy, and procedural circumstances. Third, several outcomes related to comfort, coughing, secretion burden, and willingness to undergo repeat bronchoscopy were not measured with standardized scales; therefore, we did not use these variables as formal conclusions. Fourth, total sedative doses were available, but fully standardized dose-per-kilogram-per-minute analysis could not be performed for all cases. Fifth, equipment-related differences, including scope generation, accessories, lubrication, and needle handling, may influence outcomes and should be controlled prospectively. Finally, the study was performed in a single experienced center, which may limit generalizability to units with less experience in nasal EBUS.

Despite these limitations, the study contributes useful real-world evidence regarding the route of insertion for linear EBUS-TBNA. For interventional pulmonologists, the main lesson is that the nasal route can be a practical alternative to the oral route when nasal anatomy is suitable and the team is familiar with the technique. The nasal route may be especially useful in patients in whom oral manipulation, deep sedation, or hypoxemia-related interruption is a concern.

## Conclusions

**Fig. 1:**
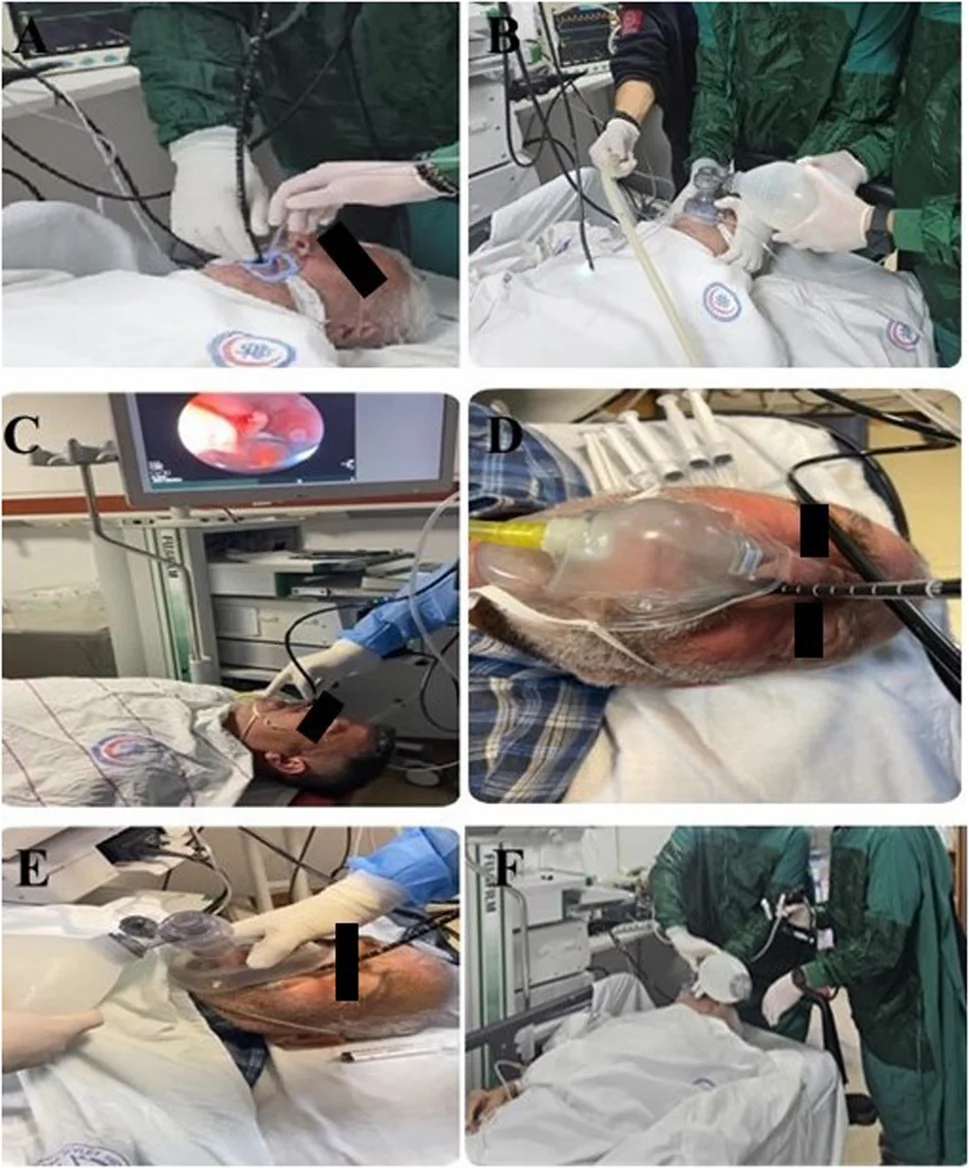
Representative procedural photographs. **A**, **B** Oral EBUS procedures performed under conscious sedation with a mouthpiece and oxygen support. **C**, **D** Nasal EBUS procedures performed under conscious sedation. **E**, **F** Airway support and rescue ventilation maneuvers during transient respiratory compromise. EBUS, endobronchial ultrasound

**Fig. 2:**
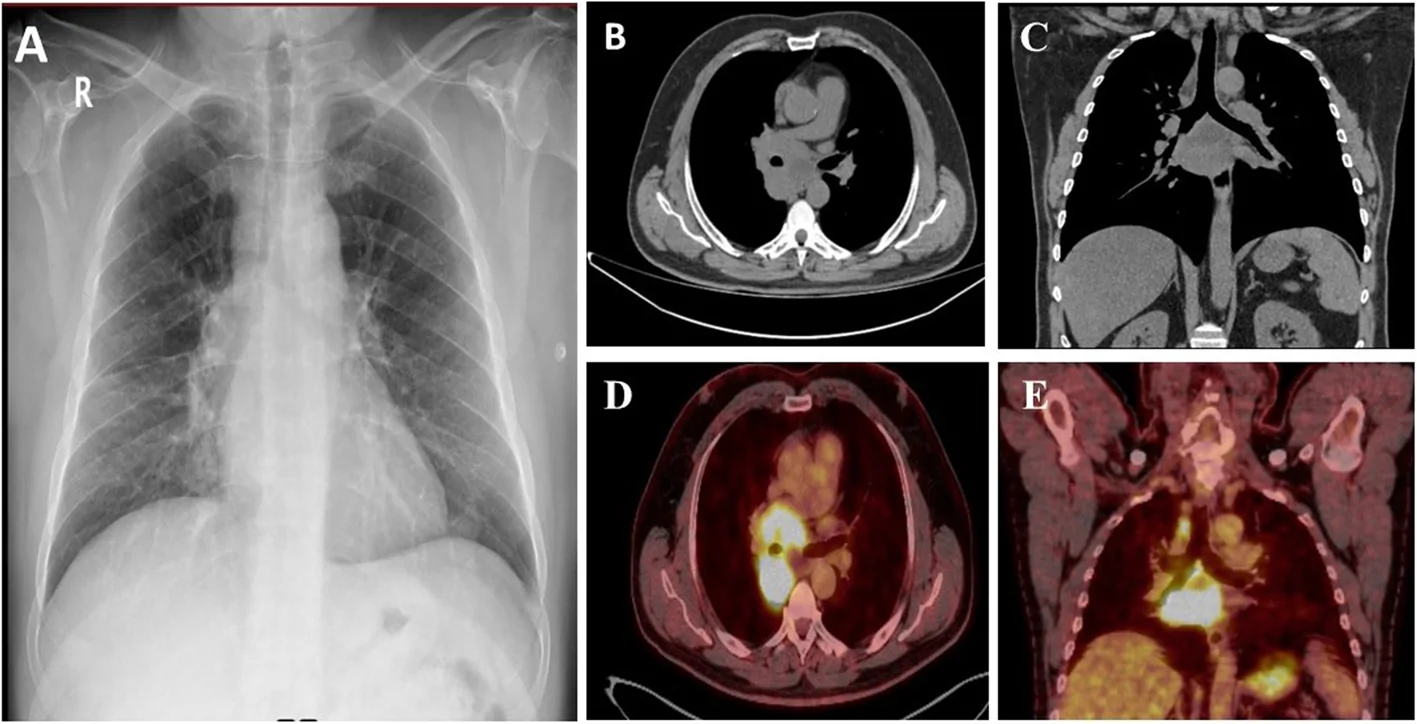
Representative imaging from an adult male patient evaluated for mediastinal and hilar lymphadenopathy and ultimately diagnosed with malignant disease. **A** Posteroanterior chest X-ray showing mediastinal widening/hilar fullness. **B**, **C** Axial and coronal chest computed tomography images showing enlarged mediastinal and hilar lymph nodes. **D**, **E** Combined positron emission tomography/computed tomography images demonstrating hypermetabolic mediastinal/hilar lymphadenopathy

**Fig. 3:**
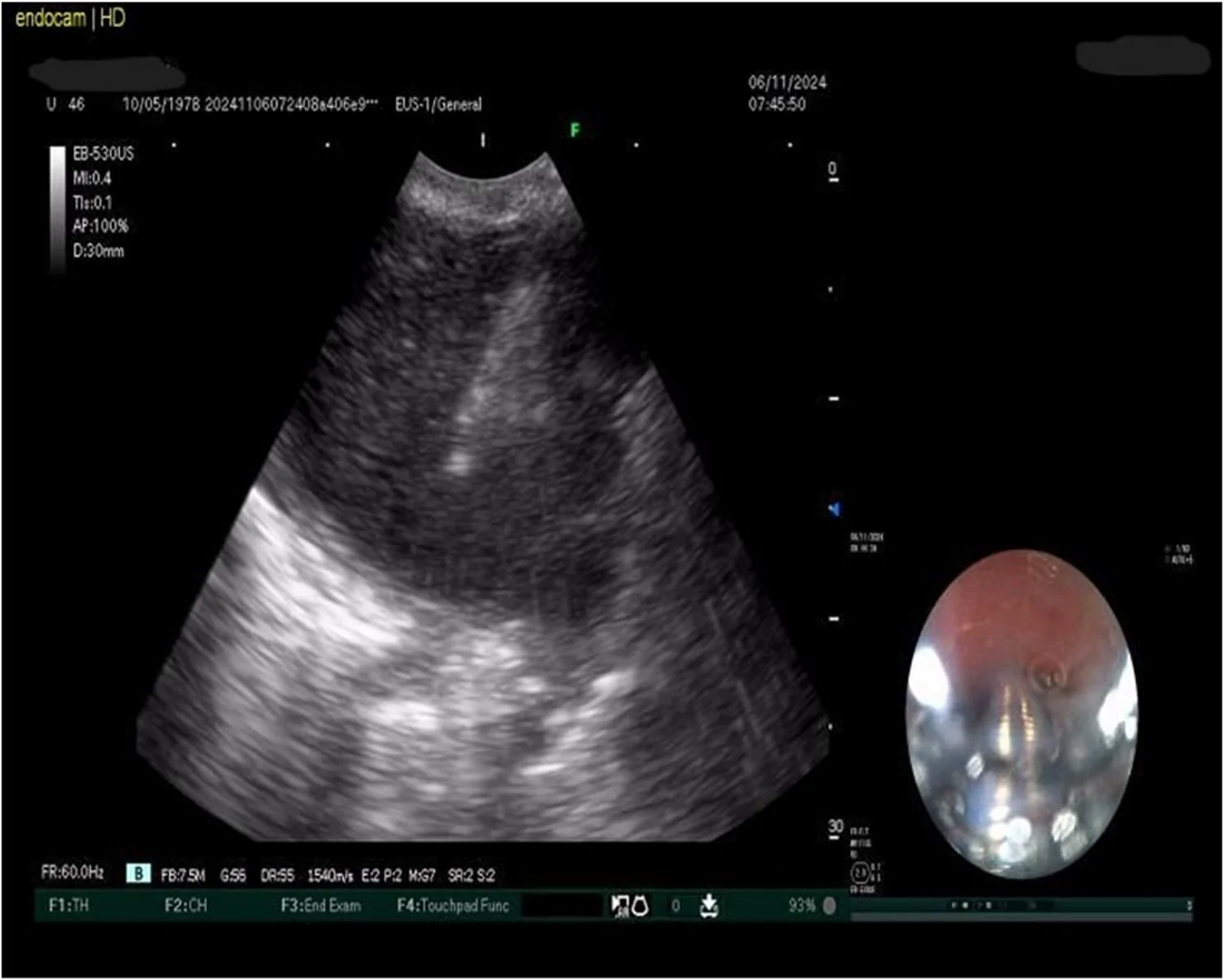
Representative image of a linear endobronchial ultrasound (EBUS) procedure showing the ultrasound image and the corresponding endobronchial view

Nasal linear EBUS-TBNA was a safe and feasible alternative to oral EBUS-TBNA in this retrospective single-center cohort. The nasal route provided comparable diagnostic results and similar major complication rates while being associated with lower sedative requirements, fewer hypoxemia-related interruptions, fewer re-EBUS procedures, and a modest reduction in procedure time. The new contribution of this work is the demonstration, in a relatively large real-world cohort, that the nasal route may improve procedural workflow without reducing diagnostic performance. Prospective randomized studies using standardized patient comfort measures, weight-adjusted sedative dosing, and detailed equipment documentation are needed to confirm these findings (Figs.1, 2 and 3).

## Data Availability

The datasets used and/or analyzed during the current study are available from the corresponding author on reasonable request.

## Abbreviations

EBUS: Endobronchial ultrasound
EBUS-TBNA: Endobronchial ultrasound-guided transbronchial needle aspiration
CT: Computed tomography
PET/CT: Positron emission tomography/computed tomography
SpO2: Peripheral oxygen saturation
HADS-A: Hospital Anxiety and Depression Scale-Anxiety subscale
SD: Standard deviation
